# The shifting balance: innate lymphoid cell plasticity in chronic gut diseases

**DOI:** 10.3389/fimmu.2026.1826115

**Published:** 2026-09-21

**Authors:** Ping Ling, JinTao Fang, JinXin Wang, YuJie Xu, NaFei Huang, Ru Mu, Yan Shen, HuaJun Zheng

**Affiliations:** 1 The Second School of Clinical Medicine, Zhejiang Chinese Medical University, Hangzhou, China; 2 Hangzhou School of Clinical Medicine, Zhejiang Chinese Medical University, Hangzhou, China; 3 School of Basic Medical Sciences, Zhejiang Chinese Medical University, Hangzhou, China; 4 The Second Affiliated Hospital of Zhejiang Chinese Medical University, Hangzhou, China

**Keywords:** functional reprogramming, immune homeostasis, innate lymphoid cells, intestinal diseases, plasticity

## Abstract

Innate lymphoid cells (ILCs), as core components of innate immunity, coordinate mucosal immune defense and homeostasis through their functional plasticity. They exhibit functional diversity in the intestine and display a complex dual role in intestinal diseases, which is closely associated with their phenotypic transformation and functional reprogramming. Current studies have demonstrated that ILCs play critical roles in various chronic intestinal diseases, including autoimmune intestinal disorders, chronic infectious intestinal inflammation, and colorectal cancer. However, the specific functions and reprogramming mechanisms of ILC subsets remain controversial across different types of intestinal diseases. This review provides a comprehensive overview of current research on the mechanisms of ILCs in chronic intestinal diseases, aiming to advance the development of immunotherapeutic strategies targeting ILC reprogramming and offer novel insights into the prevention and treatment of intestinal diseases.

## 1 Introduction

Innate lymphoid cells (ILCs) (1) are key components of the innate immune system, playing a dual role: defending against infections and maintaining internal homeostasis. Recent studies (2) have revealed that ILCs exhibit remarkable functional diversity under different physiological and pathological conditions. In particular, within the gut, ILCs not only participate in the defense against pathogenic invasion but also play a critical role in sustaining intestinal immune homeostasis (3).

Group 1 ILCs (ILC1s), Group 2 ILCs (ILC2s), and Group 3 ILCs [including ILC3s and lymphoid tissue inducer cells] represent three major subsets of ILCs, with each characterized by distinct characteristics in terms of cytokine production and immunoregulatory functions (4). The dysfunction of ILCs constitutes a critical event in the pathogenesis of intestinal diseases such as Crohn’s disease (CD) and ulcerative colitis (UC) (5–7). Studies have shown that both the number and function of ILCs are significantly altered in these conditions (8–10). Consequently, the reprogramming mechanisms of ILCs are receiving increasing attention in the onset and progression of intestinal diseases (11). This reprogramming involves extensive remodeling of cytokine expression profiles, key transcription factors, and metabolic pathways, profoundly influencing the survival and functions of ILCs. Current research suggests that ILCs can undergo functional transformation in response to external stimuli and endogenous signals within the microenvironment (12). In summary, the functional diversity of ILCs in the intestine and the mechanisms underlying their reprogramming offer new perspectives for understanding their roles in intestinal diseases. Continued investigation into the biological characteristics of ILCs and their mechanisms of action in diseases will lay a theoretical foundation for the development of novel immunotherapeutic strategies.

## 2 Classification and functions of ILCs

Innate lymphoid cells (ILCs) collectively originate from common lymphoid progenitors (CLPs) in the bone marrow. Under the instructive control of transcription factor ID2, they commit to the innate lymphoid differentiation pathway, where a critical lineage bifurcation takes place: one branch proceeds through NK cell precursors (NKp) and, driven by EOMES and T-bet, differentiates into cytotoxic conventional NK cells (cNKs), functionally analogous to CD8^+^ cytotoxic T cells; the other branch derives from common helper-like innate lymphoid cell progenitors (CHILPs) and PLZF^+^ ILC progenitors (ILCPs), generating helper-like ILCs. ILCs are collectively classified into three major functional groups: Group 1 ILCs encompass T-bet^+^ cNKs and helper-like ILC1s, with the former executing cytotoxic killing and the latter performing IFN-γ/TNF-α-mediated helper functions; Group 2 ILCs represent GATA3^+^RORα^+^ ILC2s that secrete IL-5, IL-13, and IL-9; Group 3 ILCs represent RORγt^+^AHR^+^ ILC3s (including lymphoid tissue inducer cells) that secrete IL-17A and/or IL-22. Together, these constitute a complete lineage architecture extending from a common progenitor through cytotoxic and helper-like branches to three functionally distinct subsets (**Figure 1**; **Table 1**) (1, 13).

**Figure 1:**
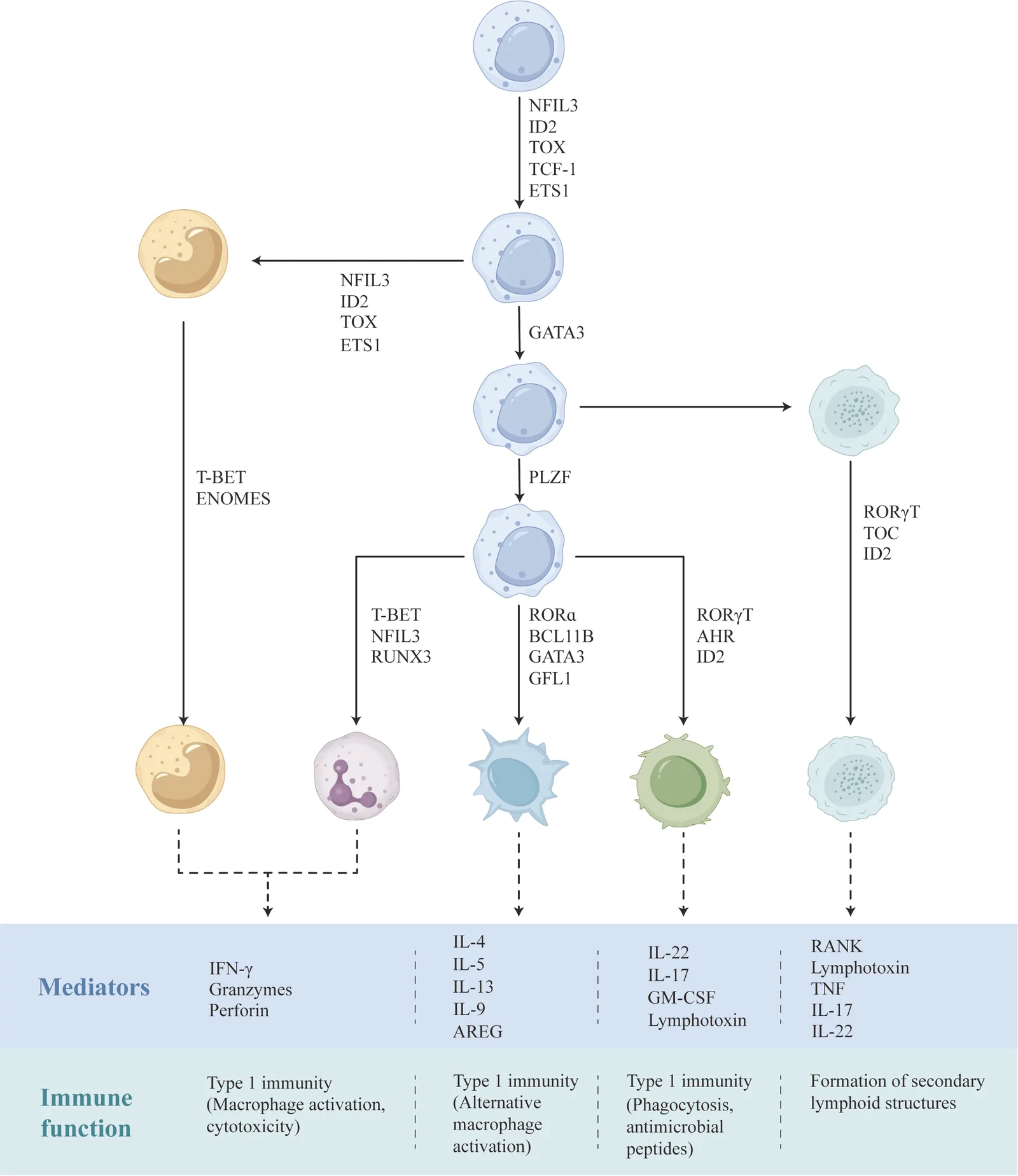
Classification and Functions of ILCs.

**Table 1:** Classification and Functions of ILCs.

ILC subsets	cNKs	Helper-like ILC1s	ILC2s	ILC3s	LTi cells
Type of ILCs	Group 1 ILC	Group 1 ILC	Group 2 ILC	Group 3 ILC	Group 3 ILC
Surface marker: MICE	NK1.1, CD49b, NKG2D, NKp46, CD122, Ly49 receptors	CD127, CD49a, CD161, NKp46	CD127, Sca-1, CD25, KLRG1, IL-17RB	CD127, CCR6, NKp46^+^/^-^, CD117, CD4^+^/^-^	CD4, CD127, CD25, CCR7, CXCR5, CD117
Surface marker: HUMANS	CD56, CD16, NKp30, NKp44, NKp46, CD94/NKG2A, CD122	CD127, CD161, NKp46, CRTH2^-^	CD127, CD161, CRTH2, KLRG1	CD127, CD161, CCR6, NKp44^+^/^-^, CD117, CD4^+^/^-^	CD4, CD127, CD25, CCR7, CXCR5, CD117
Cytokines Required For Development and Activation	IL-15 (development); IL-12, IL-18, IL-2 (activation)	IL-12, IL-15, IL-18 (activation); IL-7 (survival)	IL-25, IL-33, TSLP (activation); IL-2, IL-7 (survival/expansion)	IL-1β, IL-23 (activation); IL-7 (survival); AHR ligands (development)	IL-7 (development); RANKL–RANK signaling
Transcription factors	EOMES, T-bet (TBX21)	T-bet (TBX21)	GATA3, RORα	RORγt, AHR	RORγt, AHR
Mediators produced	IFN-γ, TNF-α, perforin, granzyme B	IFN-γ, TNF-α	IL-5, IL-13, IL-9	IL-17A, IL-22	LTα1β2, IL-17A, IL-22
Function	Cytotoxic killing	IFN-γ/TNF-α-mediated helper functions	Type 2 immunity, anti-helminth defense, allergic inflammation, tissue repair	Defense against extracellular bacteria/fungi, mucosal barrier maintenance	Secondary lymphoid organogenesis (lymph node & Peyer’s patch development)

Group 1 ILCs serve as the core executive cells of the innate immune system, comprising cytotoxic conventional NK cells (cNKs) and helper-like ILC1s. Both subsets, under the instructive control of the transcription factor T-bet, are capable of producing IFN-γ and TNF-α; however, they exhibit distinct functional specialization. cNKs rely on EOMES and the perforin/granzyme system to execute cytotoxic killing, being primarily responsible for tumor immune surveillance and direct clearance of virus-infected cells. By contrast, helper-like ILC1s (hereinafter referred to as ILC1s) predominantly reside in mucosal tissues, where they mediate defense against intracellular pathogens and regulate local inflammation through the secretion of IFN-γ and TNF-α. In the context of protective immunity, IFN-γ enhances the phagocytic function and antimicrobial activity of macrophages via activation of the STAT1 signaling pathway, making it indispensable for the clearance of intracellular pathogens (14, 15). Additionally, TNF-α, another critical effector molecule of ILC1s, induces the expression of endothelial adhesion molecules to rapidly recruit neutrophils to infection sites of acute intestinal mucosal inflammation (16), which may be a key link in the immune effect of ILC1s function. Notably, the synergistic effect of IFN-γ and TNF-α constitutes a dynamic equilibrium network: during viral infections or early-stage tumors, both cytokines exert protective effects by promoting antigen presentation and facilitating immune cell infiltration; however, under conditions of chronic inflammation, excessive release of both cytokines may disrupt homeostasis of the microenvironment, leading to a cytokine storm and tissue injury (17–19). In summary, the functional plasticity of ILC1s finely regulates the balance between infection defense and inflammatory pathology through dynamic shifts in their cytokine secretion profiles. Elucidating the tissue-specific regulatory networks underlying this process will provide a theoretical basis for achieving precise immune interventions.

Group 2 ILCs (hereinafter referred to as ILC2s) are driven by the lineage-determining transcription factors GATA3 and RORα, with IL-5 and IL-13 serving as their core effector molecules. They further induce the production of IL-9 and amphiregulin (AREG), thereby constituting a complex type 2 immune regulatory network that plays a predominant role in anti-parasite immunity, allergic inflammation, and tissue repair (20, 21). Specifically, during parasitic infection, ILC2s regulate eosinophil recruitment through the GATA3–IL-5/IL-13 axis, thereby maintaining a dynamic balance between pathogen elimination and tissue repair (22, 23). However, under pathological conditions, aberrant activation of ILC2s disrupts this balance—excessive release of IL-13 triggers inflammatory cascades by activating the STAT6 signaling pathway, leading to airway hyperresponsiveness in asthma and skin barrier dysfunction in atopic dermatitis (24, 25). Further studies indicate that ILC2s activate the JAK/STAT pathway via autocrine IL-9, which not only supports their own survival but also recruits mast cells to amplify allergic response (26). Additionally, ILC2s activate the transcription factor GATA3 via the Notch signaling pathway (Notch1-hes1), driving the secretion of type 2 cytokines and thereby promoting Th2 cell differentiation (27). Notably, the bidirectional regulatory roles of ILC2s are particularly prominent in metabolic immunity. On one hand, IL-4 secreted by ILC2s impairs immune tolerance by inhibiting the differentiation of allergen-specific regulatory T cells (Tregs) (28). On the other hand, IL-4 promotes the polarization of M2 macrophages in adipose tissue, thereby contributing to the regulation of energy metabolic homeostasis and the strengthening of the immune barrier (29). Based on the aforementioned mechanisms, targeting the activation pathways of ILC2s or their effector molecule secretion has emerged as a potential therapeutic strategy for type 2 immunity-related diseases (24, 30). However, further research is needed to fully elucidate the tissue-specific heterogeneity and plasticity of ILC2 subsets.

Group 3 ILCs (hereinafter referred to as ILC3s) are driven by the lineage-determining transcription factors RORγt and AHR. Based on natural cytotoxicity receptor (NCR) expression, they can be subdivided into three subsets: NCR^+^ ILC3s, NCR^-^ ILC3s, and lymphoid tissue inducer (LTi)-like ILC3s. Their core effector molecules include IL-22, IL-17A, and granulocyte-macrophage colony-stimulating factor (GM-CSF), and they coordinate mucosal barrier defense, tissue repair, and immune homeostasis maintenance through interactions with stromal and epithelial cells (31). Among these, LTi-like ILC3s mediate the formation of secondary lymphoid organs during embryonic development via the lymphotoxin (LT)-LTβR signaling axis (32). Specifically, IL-17 secreted by ILC3s is directly associated with neutrophils recruitment in the injured area (33), whereas IL-22, by activating the STAT3 signaling pathway, induces the secretion of antimicrobial peptides, including RegIIIγ and β-defensins, as well as the mucin MUC2 by intestinal epithelial cells, thereby strengthening the physical barrier, inhibiting pathogen colonization (34–36), and promoting epithelial repair in response to tissue injury (37). In contrast, GM-CSF exhibits dual regulatory functions. During *Candida albicans*-induced infection, GM-CSF enhances local antimicrobial activity by driving the differentiation of monocytes into inflammatory macrophages (38). However, under homeostasis, GM-CSF induces the differentiation of Tregs via the TGF-β signaling (39), thereby establishing immune tolerance, although Treg differentiation induced by RORγt^+^ APCs (antigen-presenting cells) may play a more dominant role, the contribution of ILC3-induced Treg differentiation should not be overlooked. In summary, by releasing the aforementioned cytokines in a sequential and balanced manner, ILC3s play a central role in the body’s resistance to infection, barrier reconstruction, and inflammation regulation. Disruption of this balance can lead to ILC3 dysfunction, which can trigger mucosal barrier disruption, and chronic inflammation (40, 41).

It is worth noting that ILCs possess cross-functional regulatory capabilities. For instance, ILC2s link immune responses to energy metabolism via the SUCNR1 signaling-mediated succinate sensing signaling pathway (42), expanding the metabolic regulation dimension of immune homeostasis. Meanwhile, ILC3s can induce the differentiation of Tregs through MHC-II-mediated antigen presentation (43, 44), highlighting their novel role as a bridge between innate and adaptive immunity. Additionally, inter-transformation and functional reprogramming among ILC subsets provide a mechanistic framework for understanding immune imbalances in inflammatory diseases.

## 3 Cell plasticity and functional reprogramming of ILCs in the immune and tumor microenvironments

Despite their highly specialized functions, the three ILC subsets exhibit remarkable phenotypic plasticity and Functional Reprogramming within inflammatory and tumor microenvironments. Cellular phenotypic plasticity denotes the biological property whereby differentiated cells, upon stimulation by internal and external environmental signals, dynamically alter their functional output and molecular signatures through reversible transcriptional reprogramming, and achieve bidirectional transitions between distinct phenotypic states under specific conditions. Functional reprogramming under intense pathological stimuli refers to a process in which the disease microenvironment, through the synergistic action of sustained and high-intensity inflammatory cytokines, hypoxic signals, and metabolic stress, compels cells to breach their established gene expression homeostasis, thereby triggering the replacement of lineage-determining transcription factors and profound rewiring of core metabolic programs. Such restructuring is not merely a reversible functional bias, namely polarization, but rather results in a qualitative alteration of cellular functional output, manifested as the acquisition of novel functional capacities or the loss of original core functions. Unlike directed interventions such as pharmacological treatment or genetic editing, pathological microenvironment-driven reprogramming lacks predetermined purposefulness, and its outcomes exhibit a marked double-edged effect: it may either perpetuate unresolved inflammatory responses or, under certain conditions, activate tissue-protective phenotypes. This remarkable plasticity and functional reprogramming endow them with an “immune adaptability” capacity that positions them as critical switches in the regulation of inflammatory balance (45) and key players within both the immune and tumor microenvironments (46, 47). The activity and functional state of ILCs are highly regulated by an integrated network of signals within the local microenvironment, including cytokines, metabolic products, and intercellular interactions. During infection or acute inflammation (47, 48), ILCs rapidly respond to pathogen- or damage-associated signals by differentiating into pro-inflammatory subsets that secrete pro-inflammatory factors such as IFN-γ and IL-17, or IL-22 to enhance immune response against pathogens and promote barrier repair. However, under the conditions of chronic inflammation (49, 50), overactivation of these pro-inflammatory subsets and the resulting pro-inflammatory cytokine storm can lead to progressively sustained local tissue injury. Moreover, within the tumor microenvironment (51, 52), ILCs may undergo phenotypic transformation or functional suppression, and in some cases even transform into pro-tumor phenotypes.

ILC reprogramming is defined as a biological process in which innate lymphoid cells, driven by microenvironmental metabolic cues or inflammatory cytokines, undergo replacement of lineage-determining transcription factors, thereby achieving qualitative changes in functional output. This process is driven by an integrated signaling network acting synergistically (**Figure 2**). First, the cytokine network serves as the core driving force: pro-inflammatory cytokines such as IL-12, IL-18, and IL-23 promote the expansion and inflammatory functions of ILC1s and ILC3s (53, 54), whereas anti-inflammatory cytokines like IL-10 and TGF-β induce the differentiation of ILC2s and the secretion of associated cytokines such as IL-4 and IL-13, thereby facilitating immune tolerance and tissue repair (55). Second, changes in environmental metabolites significantly influence the metabolic adaptation and functional phenotype of ILCs. These cytokines and metabolic signals do not merely elicit immediate functional responses; Rather, they drive ILC reprogramming through a hierarchical regulatory network. Mechanistically, pro-inflammatory cytokines, TGF-β and other immunomodulatory signals, as well as short-chain fatty acids and aberrant metabolites in the tumor microenvironment, all converge to activate specific signaling pathways, thereby inducing a switch in the expression profile of lineage-determining transcription factors (56–63).

**Figure 2:**
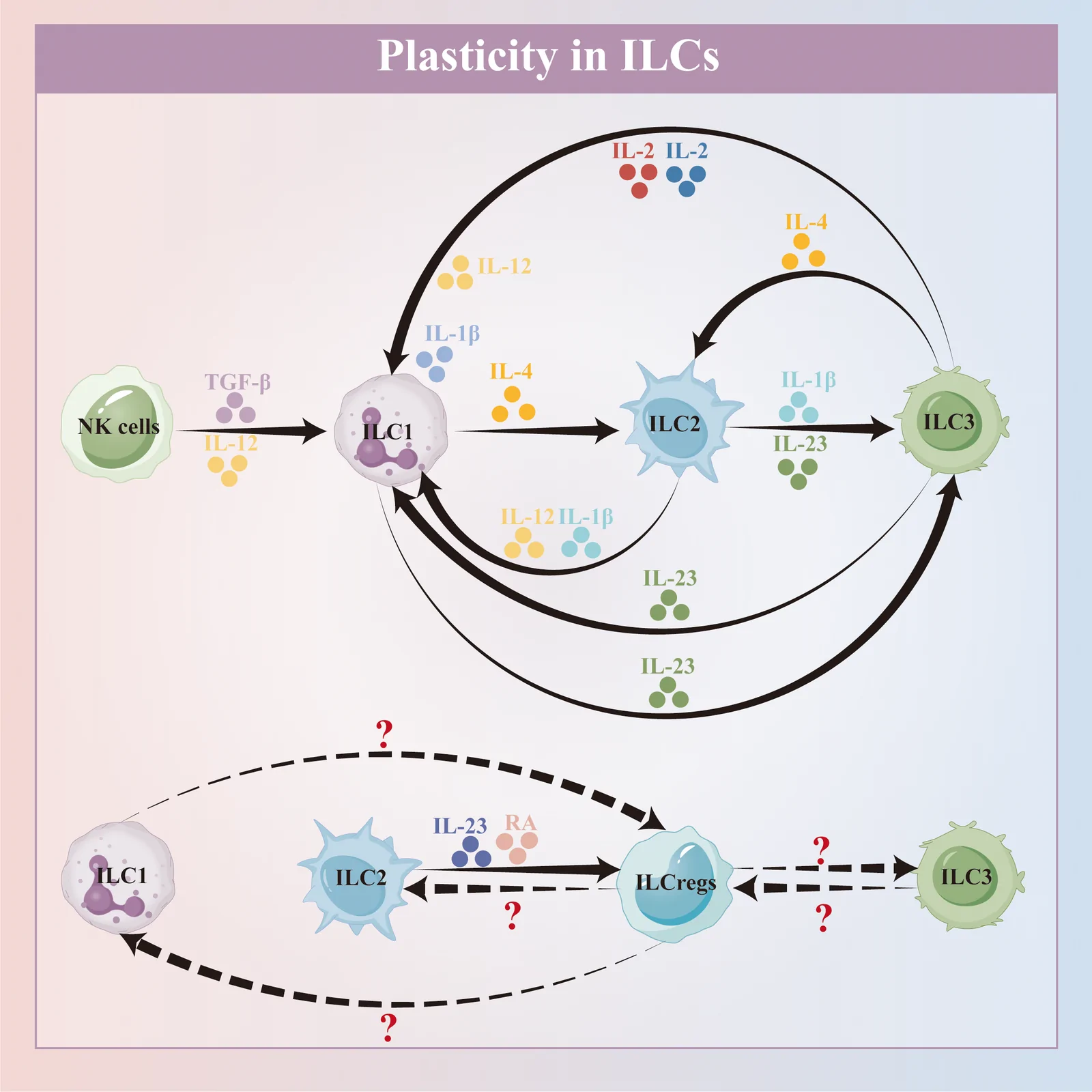
The different stimulatory axes of each ILC subsets. The symbol (?) indicates that no definitive and adequate evidence is currently available to elucidate this part of the research content/molecular mechanism.

In summary, the dynamic remodeling of ILCs within the immune and tumor microenvironments is the core driving force for the exertion of their immune functions. During infection or acute inflammation, ILCs adopt pro-inflammatory phenotypes to sustain immune defense and tissue repair; however, in chronic inflammation or tumors, their phenotypic transformation drives the dysregulation of immune responses, leading to pathological damage or immune evasion.

## 4 Distribution and functions of ILCs in the intestine

Within the intestinal mucosal immune system, ILC subsets exhibit region-specific distributions and act collaboratively to maintain barrier homeostasis. ILC1s are predominantly enriched in both the intestinal epithelium and lamina propria (64), where they activate innate immune responses against intracellular pathogens through the secretion of IFN-γ and TNF-α. ILC2s localize primarily in the deeper layers of the lamina propria, with their distribution closely overlapping with the secretion sites of epithelial-derived alarmins such as IL-25 and IL-33 (65), enabling rapid responses to tissue damage. For instance, during helminth infection, ILC2s secrete IL-13 to induce goblet cell hyperplasia for enhancing the mucus barrier, release amphiregulin to promote epithelial repair, and stimulate smooth muscle contraction to accelerate parasite expulsion (66). ILC3s predominantly accumulate around the intestinal crypts and Peyer’s patches (67), where they maintain intestinal barrier integrity by secreting IL-22 to directly induce Paneth cells to produce antimicrobial peptides such as RegIIIγ and stimulate goblet cells to synthesize the mucin MUC2 (68). Additionally, ILC3s promote the development of isolated lymphoid follicles through lymphotoxin (LTα1β2) (69), thereby providing structural support for adaptive immune responses. In summary, through their multilevel spatial distribution and functional synergy, ILCs serve as core effector cells regulating the dynamic balance of the intestinal barrier. However, the threshold between homeostasis maintenance and pathological transformation is under the precise regulation of the microenvironmental signaling network, with imbalance potentially leading to breakdown of immune tolerance and disease progression.

## 5 Roles of ILCs in the intestine and the consequences of their deficiency or hyperactivation

Loss of ILC function leads to the breakdown of immune defense and facilitates immune escape of tumor cells, whereas hyperfunction of ILCs can trigger abnormal immune responses that induce chronic intestinal inflammation and even carcinogenesis. On one hand, quantitative and functional loss of ILCs significantly weakens the host’s immune surveillance against pathogens and tumor cells. For example, a study by Burrows K et al. demonstrated that a sharp decline in the number of ILC3s markedly increases host susceptibility to *Citrobacter rodentium* infection (70). In another study, Gao Y’s research team reported that TGF-β-driven conversion of NK cells into ILC1-like phenotype incapable of regulating local tumor growth and metastasis represents a critical part of tumor escape from innate immune surveillance (71). ILC2 deficiency disrupts the homeostasis of the IL-4^+^ ILC2 subset, resulting in a reduction of IL-25^+^ tuft cells, mucosal barrier damage, and an increased tumor burden (72). On the other hand, excessive activation of ILC subsets drives chronic inflammation through aberrant immune responses and promotes inflammation-to-cancer transition via dysregulated cytokine networks. Using single-cell RNA sequencing, Saul D et al. found that ILC1s are increased in the intestines of patients with CD compared to healthy individuals, and that the inducible genes specifically upregulated in these ILC1s are also enriched in the intestines of patients with colorectal cancer (CRC). Notably, the expression levels of these genes progressively increase from a healthy state to an inflammatory state and then to a tumor state, suggesting that ILC1s may drive inflammation-to-cancer transition by mediating chronic inflammation (73). Excessive activation of the IL-25-driven ILC2-MDSC axis promotes IL-17 secretion through enhanced ILC3/Th17 responses, directly activates fibroblasts, and drives intestinal fibrosis (72). Its tumor-promoting mechanisms primarily depend on an IL-25-induced Th2-type microenvironment, which subsequently recruits myeloid-derived suppressor cells (MDSCs) to suppress CD8^+^T cell activity and IFN-γ secretion, ultimately accelerating tumor progression (74, 75). ILC2s accelerate tumor invasion by inducing a Th2-type immune microenvironment (74, 75) that suppresses anti-tumor T cell and TNF-γ responses (76). Clinical evidence further demonstrates that high IL-25 expression in tumor tissues of colorectal cancer (CRC) patients is significantly associated with reduced survival rates, and that the IL-25-ILC2-MDSC axis drives the formation of an immunosuppressive microenvironment (77). Likewise, in a mouse model of CRC, ILC3s that secrete the pro-inflammatory factor IL-17 significantly increase in number under the combined induction with AOM and DSS, with their number and function positively correlating with tumor progression (78). Furthermore, Bai X et al. revealed that aberrant activation of ILC3s drives pro-tumor STAT3 signaling and disrupts immune homeostasis through the microbiota-ILC3 axis, facilitating tumorigenesis (79).

However, certain existing evidence also suggests that ILC2s may have beneficial roles in inflammation-to-cancer transition in the intestine. Its anti-tumor functions are mediated through multiple mechanisms: direct activation of CD8^+^ T cells via IL-9 secretion (76), enrichment of the SLAMF1^+^ ILC2 subset in tertiary lymphoid structures (TLS) to promote B cell maturation and enhance anti-tumor immunity (80, 81)and the abundance of this subset is positively correlated with favorable prognosis in CRC patients (77)or through the conversion of ILC3s into cytotoxic ILC1/NK-like cells to mediate direct killing (80). Another study further supports this view, reporting that ILC2 deficiency significantly increases tumor burden in mice, and that the abundance of ILC2-associated gene signatures within tumors correlates positively with favorable prognosis in patients with CRC, suggesting that agonist-based therapies targeting ILC2s may represent a promising therapeutic strategy for improving clinical outcomes (82).

The aforementioned findings suggest that the dynamic balance and functional integrity of intestinal ILC subsets are crucial for maintaining the intestinal immune homeostasis and barrier. Disruption of this balance can not only result in impaired intestinal immunity but also lead to chronic inflammation and even promote intestinal carcinogenesis.

## 6 Relationship between ILCs and intestinal diseases

Intestinal diseases refer to a broad spectrum of conditions characterized by pathological involvement of the intestinal structure or dysfunction of intestinal physiology, encompassing a variety of types including inflammation, infection, and malignancies. This broad category includes diseases such as inflammatory bowel disease (IBD), irritable bowel syndrome (IBS), celiac disease (CeD), and CRC. The incidence and prevalence of these diseases have been rising globally, imposing a significant burden on public health worldwide (83–85). Due to their complex etiology, high recurrence rate, and the limitations of conventional treatments, elucidating the pathogenic mechanisms of intestinal diseases holds significant scientific and practical value for the development of novel targeted therapies and improvement of clinical outcomes. ILCs, as a critical class of immune regulatory cells, play a central role in maintaining intestinal homeostasis and defending against pathogens. Recent studies have revealed that dysfunction of ILCs is closely associated with multiple intestinal diseases, with alterations in their number or activity directly contributing to disease progression (78, 86). Investigating the dynamic changes of ILCs and their regulatory networks in these diseases not only provides novel insights into the pathogenesis of these diseases but also lays a theoretical foundation for developing innovative therapies based on the regulation of the immune microenvironment (**Figure 3**).

**Figure 3:**
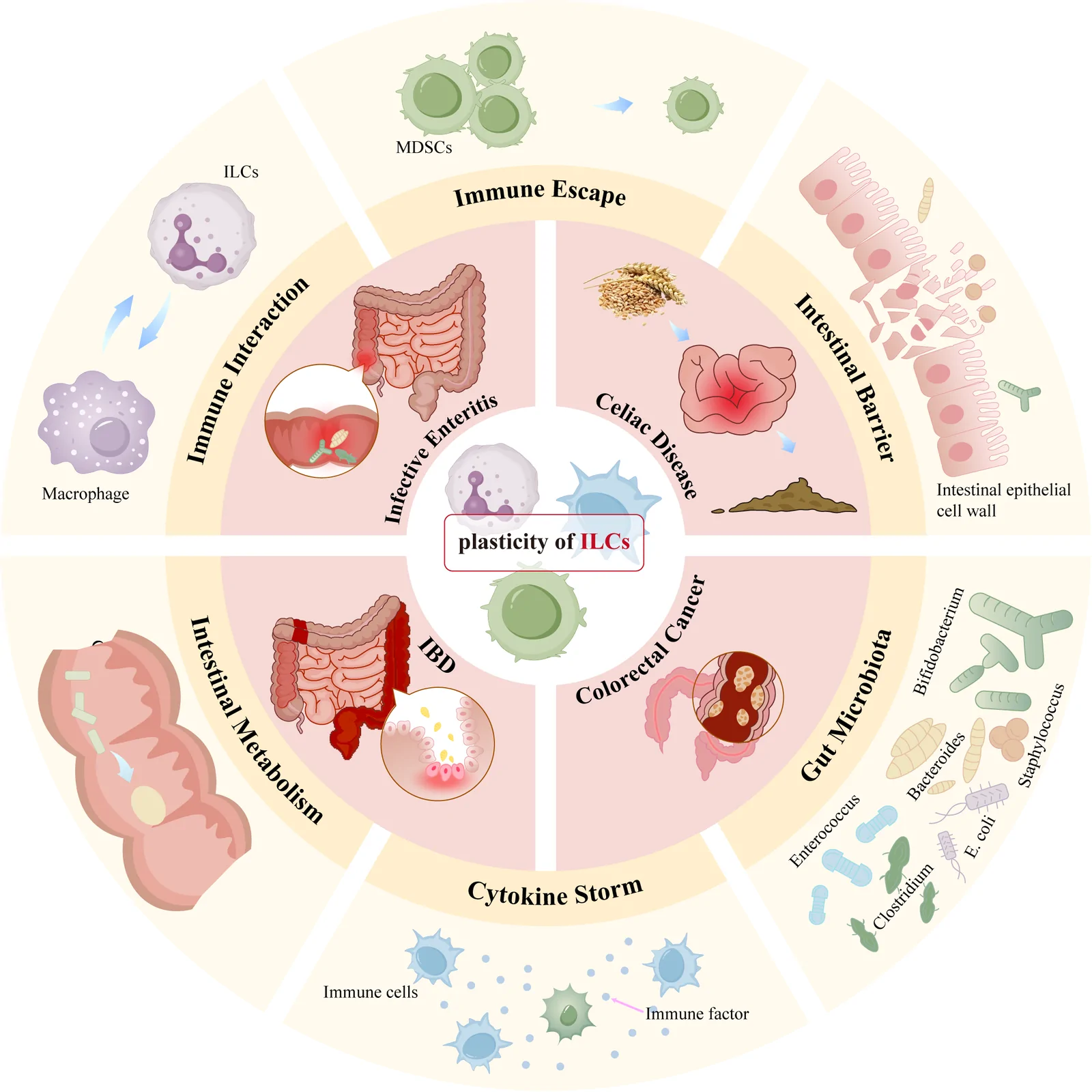
Relationship between ILCs and intestinal diseases.

### 6.1 Mechanisms of ILCs in infectious enteritis

Infectious enteritis (87) refers to acute or chronic inflammatory diseases caused by the invasion of the intestinal tract by pathogenic microorganisms such as bacteria, viruses, parasites, or fungi. Its pathogenesis is related to direct invasion of the intestinal mucosa by pathogens, toxin release, or the induction of aberrant immune responses (88). ILCs coordinate immune defense and tissue repair through multidimensional mechanisms during the pathological process of infectious enteritis. On one hand, upon pathogen invasion of the intestinal tract, ILCs, as rapid responders of the innate immune system, directly participate in anti-infective responses by secreting specific cytokines. In bacterial infections, ILC1s and ILC3s play dominant roles. McCulloch TR et al. found that, during Salmonella-induced infectious enteritis, NK cells undergo plastic differentiation into ILC1s mediated by TGF-β signaling, which enhances their pro-inflammatory function and antibacterial immune response (9). Additionally, Wang J et al. reported that ILC3s secrete IL-22 to reinforce intestinal mucosal barrier function, induce IECs to produce antimicrobial peptides and promote tissue repair, thereby directly inhibiting Salmonella colonization and invasion (89). In viral enteritis, NK cells play a core anti-viral defensive role by mediating immunomodulation through rapid early killing of infected cells and secretion of interferons. Functional impairment of NK cells can lead to delayed viral clearance in the gut and exacerbated inflammation (90). In response to parasitic infections, ILC2s serve as frontline defenders. Meiners J et al. reported that ILC2s proliferate in response to IL-33 signaling and secrete IL-9, which activates intestinal mast cells to directly mediate rapid immune responses that facilitate parasite expulsion from the gut (91). Furthermore, the anti-parasitic function of ILC2s is closely linked to their metabolic patterns. It has been reported that, during helminth-induced infectious enteritis, ILC2s maintain their function preferentially through fatty acid (FA) metabolism, and under nutrient-limiting conditions such as vitamin A deficiency, they enhance FA uptake and metabolism to selectively sustain IL-13 production. This metabolic adaptation enables ILC2s to cope with environmental stress and ensures sustained barrier immunity (92). On the other hand, ILCs maintain immune balance through interactions within their own subsets and dynamic crosstalk with other immune cells. For example, Tregs secrete IL-10 to suppress the activation of ILC1s and ILC3s, thereby alleviating inflammation (93); ILC3s modulate T cell differentiation via antigen presentation (94); and the balance of activity among different ILC subsets influences the resolution of inflammation or progression to chronicity in the later stages of infection (13). These mechanisms highlight the central role of ILCs in infectious enteritis and provide a theoretical basis for developing immunomodulatory therapies targeting ILCs. However, their functional heterogeneity and microenvironmental dependence require further investigation to optimize clinical translation strategies.

### 6.2 Mechanisms of ILCs in IBD

IBD (95), encompassing Crohn’s disease and UC, is a group of chronic inflammatory, immune-mediated, systemic diseases primarily affecting the colon and rectum. Clinically, IBD is characterized by recurrent relapses, a prolonged and refractory disease course, and susceptibility to various complications such as opportunistic infections (96). Currently, the imbalance of intestinal immune homeostasis—marked by abnormal networked interactions among immune components—is widely recognized as a core factor inducing IBD onset and driving disease progression. However, a comprehensive understanding of its regulatory mechanisms has yet to be fully established (97). With the progressive elucidation of ILC lineages (1), increasing attention has been paid to their roles in immune-related diseases. In recent years, accumulating evidence has demonstrated that ILCs play key roles in the immunopathogenesis of IBD (98). First, functional imbalance within ILC subsets directly drives intestinal inflammation and barrier disruption. Under healthy conditions, ILC1s, ILC2s, and ILC3s maintain intestinal homeostasis by secreting specific cytokines (99). However, such homeostatic balance is profoundly disturbed in patients with IBD (100). For example, ILC1s play a dominant role in the Th1-type immune response of Crohn’s disease by persistently secreting IFN-γ and TNF-α, driving intestinal Th1-type inflammation and promoting epithelial barrier damage and tissue destruction (101, 102). In contrast, ILC3s exhibit a dual role in pathogenesis: although IL-22 secreted by ILC3s is indispensable for epithelial repair (10, 36, 103), dysbiosis of intestinal flora or excessive IL-23 signaling can make them abnormally produce IL-17A, which directly impairs the mucosal barrier and promotes fibrosis (104, 105). The paradoxical functions of ILC2s also warrant attention. While their secretion of IL-13 enhances mucus secretion, it may simultaneously aggravate intestinal wall fibrosis by activating fibroblasts (106). Such subset-specific functional dysregulation is a key factor in sustained chronic inflammation in IBD. Notably, clinical reports suggest that biological therapies for IBD may exert therapeutic effects by modulating the composition of ILC subsets within the intestinal mucosa (107). Second, the gut microbiota indirectly regulates ILC activity through metabolites and immune signaling, forming a microbiota-ILCs-inflammation axis. On one hand, intestinal commensal bacteria produce SCFAs (e.g., butyrate), which activate G protein-coupled receptors to enhance the protective functions of ILC3s, promoting epithelial regeneration and antimicrobial peptide expression (108, 109). On the other hand, pathogenic bacteria like adherent-invasive *Escherichia coli* can exacerbate tissue damage by excessively activating the IL-17A pathway in ILC3s (110). Additionally, microbiota-derived tryptophan metabolites regulate ILC3 differentiation through the AHR, further influencing their transformation toward a pro-inflammatory phenotype (10, 111). Third, ILCs amplify inflammatory cascades by forming dynamic interaction networks with other immune cells, either synergistically or antagonistically. ILC3s and Th17 cells share a common IL-23-dependent activation pathway and act synergistically via IL-17A to recruit neutrophils and damage epithelial integrity (112, 113). Furthermore, ILC3s present antigens through MHC class II molecules, inhibiting the differentiation of CD4+ T cells into Tregs, thereby impairing immune tolerance (114). This imbalance in intercellular communication is a critical reason why IBD is difficult to resolve spontaneously. In summary, targeting the plasticity of ILCs—such as inhibiting NK cell-to-ILC1 conversion via blockade of TGF-β signaling—offers novel avenues for the regulation of inflammation. However, ILC functions are highly dependent on microenvironmental cues, and the boundary between their pro-inflammatory and protective roles remains blurred (**Figure 4**). Therefore, future research should integrate single-cell multi-omics and spatial transcriptomics (115) to precisely characterize the molecular features and regulatory nodes of ILC subsets at different stages of IBD, ultimately enabling personalized therapy.

**Figure 4:**
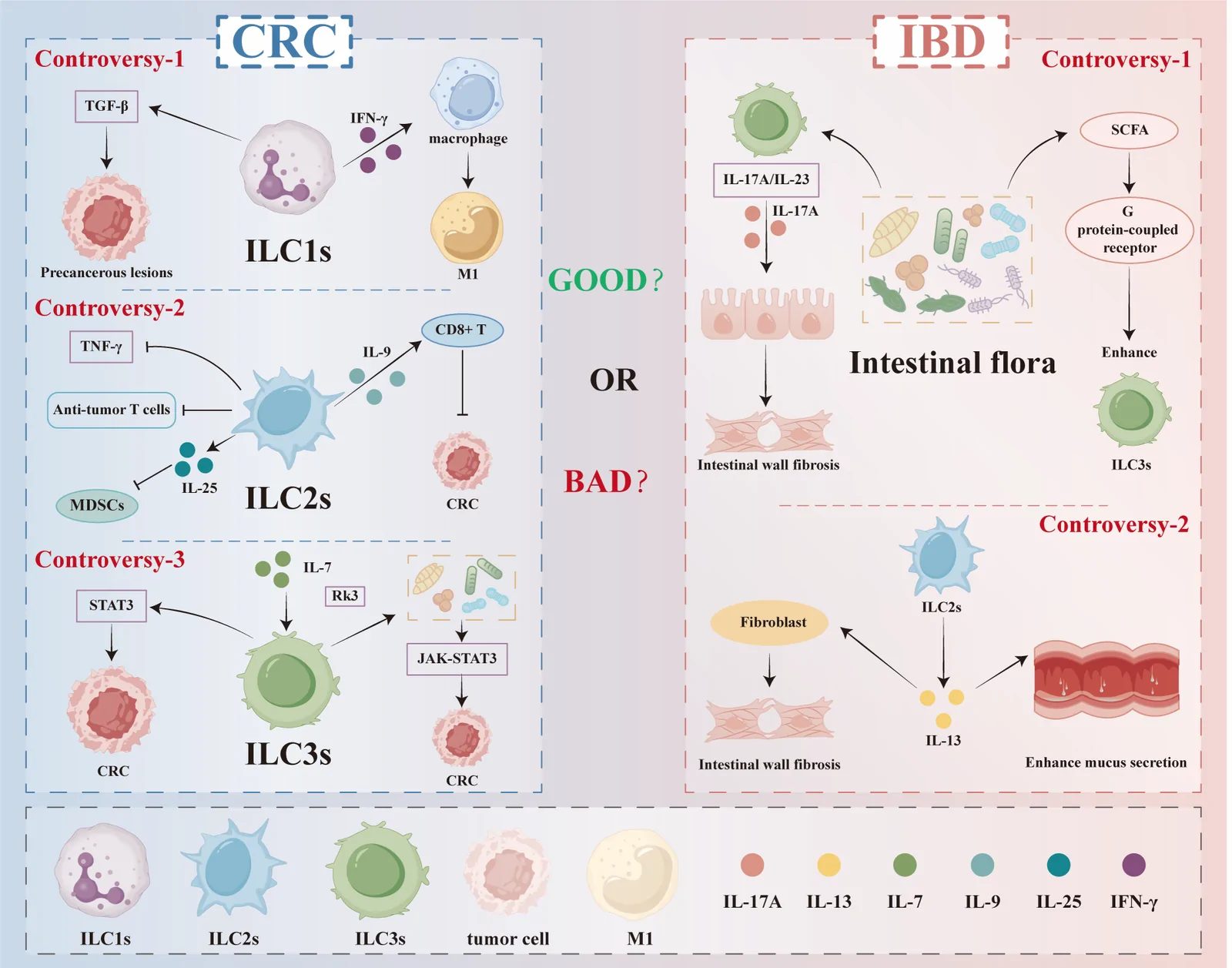
The specific functions and proportion of ILC subsets remain controversial across IBD and CRC.

Although existing evidence has established the pivotal roles of ILCs in the pathological progression of IBD, several core controversies and knowledge gaps continue to constrain their translation into clinical applications. The primary issue lies in the fact that the functional duality of ILC subsets is not a static attribute but rather dynamically shifts across disease stages: ILC3s that exert protective effects during the acute phase may transform into pro-inflammatory phenotypes in the chronic phase; however, the spatiotemporal patterns governing this transition and the boundaries of its reversibility remain unsupported by direct evidence from longitudinal lineage tracing. Second, current targeted strategies struggle to distinguish between the pro-inflammatory and protective outputs of ILCs. For instance, blockade of IL-23 may suppress IL-17A production by ILC3s while simultaneously compromising IL-22-mediated epithelial repair, and this “double-edged sword” effect poses a significant challenge to precise intervention. Third, mechanistic studies to date have relied predominantly on murine models, yet intestinal ILCs in human IBD patients exhibit marked interspecies differences in phenotypic markers, tissue-residency characteristics, and microbiota interaction patterns, rendering the clinical extrapolation of animal experimental conclusions uncertain. Furthermore, whether the modulation of ILC subset composition by biologics represents a primary therapeutic mechanism or merely an accompanying phenomenon remains to be confirmed through cell-specific intervention experiments that establish the causal chain. Therefore, future research must move beyond static phenotypic descriptions and integrate humanized models, spatial transcriptomics, and dynamic lineage tracing technologies to precisely deconstruct the molecular switches governing ILC functional conversion in a spatiotemporal dimension, thereby defining the feasible boundaries and risk thresholds for ILCs as therapeutic targets in IBD.

### 6.3 Mechanisms of ILCs in CeD

CeD (116) is a chronic autoimmune disorder triggered by gluten ingestion in genetically susceptible individuals. It is characterized by HLA-DQ2/DQ8–mediated presentation of gluten-derived peptides, the production of tissue transglutaminase autoantibodies, and villous atrophy of the small intestine. As core regulators of intestinal innate immunity, ILCs are involved in the pathogenesis of CeD by regulating immune polarization and epithelial barrier integrity (117–119). Marafini et al. (120) demonstrated that TNF-α- and IFN-γ-producing ILC1s are abnormally enriched in the intestinal mucosa of patients with CeD, where they are activated via the TLR3-poly I:C signaling pathway. This activation markedly enhances the release of inflammatory cytokines, which directly contribute to epithelial injury and villous atrophy in the small intestine. Specifically, upon pathogen recognition via TLR3, ILCs initiate a TNF-α cytokine storm through the TRIF/IRF3 pathway, which synergize with IFN-γ to enhance antigen presentation, thereby exacerbating gluten-induced autoimmune responses. Evidence from the animal models of CeD further confirms that depletion of ILCs blocks poly I:C-induced intestinal injury, establishing ILCs as central effector cells in TLR3-dependent inflammation in CeD. Similarly, Uhde M et al. (119) reported that cytotoxic NKp44+ ILC1s are decreased while pro-inflammatory NKp44-ILC1s are increased in the small intestinal epithelium of patients with CeD. The latter subset exacerbates mucosal damage through the release of IFN-γ and cytolytic granules, with their abundance positively correlating with disease severity. Restoration of NKp44+ ILC1s after dietary intervention suggests their potential as a biomarker for disease activity. Moreover, ILCs may exert their functions by regulating the activity of other immune cells. Ercolano et al. (121) reported an abnormal expansion of ILC precursors in the peripheral circulation of patients with CeD, which, under stimulation by gluten-derived peptides (p31-43/pα-9), contribute to the formation of a pro-inflammatory microenvironment through the secretion of IL-18 and IFN-γ, and upregulate transglutaminase 2 expression, thereby promoting CD4+ T cell activation. It is noteworthy that clinical overlap exists between CeD and IBS, specifically non-celiac gluten sensitivity (NCGS). Although the functional heterogeneity of ILCs in these two diseases remains to be fully elucidated, current evidence indicates that ILC1s in the rectal mucosa of patients with non-celiac wheat sensitivity contribute to inflammatory responses by secreting IFN-γ, and their numbers significantly increase following wheat exposure and reversibly decrease after a gluten-free diet. Moreover, they infiltrate the mucosa in concert with CD3^+^ T cells (122) and are involved in the pathogenesis of NCGS. In summary, ILCs participate in the core pathological processes of CeD through a multidimensional immune regulatory network. However, the overall body of evidence remains limited, and the molecular mechanisms underlying their interplay with IBD require further in-depth investigation.

### 6.4 Mechanisms of ILCs in CRC

CRC (123) refers to a malignant tumor that originates from the epithelium of the colon or rectum. Its onset and progression result from the interplay of multiple factors, including genetic mutations, epigenetic dysregulation, chronic inflammation, and gut microbiota imbalance (124, 125). As key effector cells of the innate immune system, ILCs play a double-edged role in the pathogenesis of CRC by dynamically regulating inflammatory responses, tissue repair, and the balance of the immune microenvironment (81, 126). A single-cell sequencing analysis has identified tumor-specific subsets of ILC1s and ILC2s in patients with CRC (81). ILC1s activate classical antitumor immune responses by secreting IFN-γ and TNF-α (127); however, their overactivation may disrupt the integrity of the intestinal epithelial barrier and promote chronic inflammation mediated by the TGF-β signaling pathway, thereby facilitating the formation of precancerous lesions (128). However, studies have also indicated that in CRC, ILC1s secrete IFN-γ to promote macrophage polarization toward the antitumor M1 phenotype, thereby inhibiting tumor progression (129). ILC2s in CRC exhibit marked microenvironment-dependent polarization, with their pro- or anti-tumorigenic phenotypes precisely governed by the dynamic interplay of disease stage, microbiota status, and cytokine networks. In IL-25-rich advanced microenvironments, ILC2s recruit MDSCs via IL-13 secretion through the formation of an “IL-25-ILC2-MDSC axis” (74, 75), directly suppressing CD8^+^ T cell function and accelerating tumor progression. Conversely, in early-stage lesions with stable microbiota, the IL-33-induced SLAMF1^+^ ILC2 subset (80) exerts anti-tumor effects through dual mechanisms: secreting IL-9 to activate CD8^+^ T cell cytotoxicity (76), and organizing tertiary lymphoid structures (TLS) to recruit CXCL13^+^ B cells, thereby promoting IgA^+^ plasma cell differentiation and antibody production. The apparent discrepancy between the studies by Zhao and Wan is thus reconciled: the pro-tumorigenic effect observed in the former stems from ILC2-MDSC axis activation under late-stage IL-25 overload, whereas the protective effect identified in the latter is mediated by the early IL-33-driven SLAMF1^+^ subset, with both representing plastic manifestations of the same cell population under spatiotemporal heterogeneity. Lin Y et al. further showed that ILC2s suppress the development of colitis-associated CRC by inhibiting epithelial STAT6 activation in a microbiota-dependent manner via the secretion of IL-13 (130). Notably, sustained IL-25 stimulation drives ILC2 transformation into a pro-fibrotic phenotype (74), synergizing with the ILC3/Th17 axis to mediate IL-17-dependent collagen deposition and accelerate tumor invasion. Under physiological conditions, ILC3s maintain gut microbial homeostasis through MHC class II-mediated interactions with T cells. Dysfunction of ILC3s leads to adaptive immune dysregulation within the tumor microenvironment, promoting the progression of CRC and resistance to immunotherapy (94). Moreover, Javadzadeh SM et al. observed a significant increase in the proportion of ILC3s in the *in situ* dysplastic lesion group of a CRC mouse model, where they promoted tumor progression through secretion of pro-inflammatory cytokines and remodeling of the tumor microenvironment (78). Additionally, existing studies indicate that ILC3s are regulated by IL-7-mediated AHR and Card9/IL-1β-dependent pathways, which activate STAT3 signaling to enhance the secretion of IL-22, thereby contributing to the development of CRC (58, 131). In contrast, Wu J et al. reported a negative correlation between ILC3s and the pathological stages in colon cancer (132). Bai X et al. also found that, through Rk3-mediated modulation of the gut microbiota, ILC3s suppress activation of the JAK-STAT3 pathway and the expression of Th17-related pro-inflammatory factors, thereby inhibiting the development of colon cancer. Others have demonstrated that in CRC, ILC3s are reduced while ILC1s are increased, indicating a functional shift in the ILC compartment (94, 126). However, this transition is not abrupt or complete. Alessia Calabrò et al. Identified a population of ILC3s in murine models that acquire NK-like features and respond to IL-12 stimulation, yet continue to express RORγt. These transitional cells—often referred to as NKp46^+^ LTi-like cells—retain the capacity to produce lymphotoxins and adhesion molecules, thereby contributing to the maintenance of lymphoid architecture and facilitating antitumor immune responses (133). Currently, there remain gaps in understanding the functional heterogeneity of ILCs in CRC, and the molecular switches governing phenotypic transformation among ILC subsets are yet to be fully elucidated (**Figure 4**). In summary, ILCs are involved in the pathological process of CRC through complex regulatory networks. However, the functional plasticity of ILCs and the complexity of their interactions with the microenvironment necessitate future research to integrate technologies such as single-cell sequencing and spatial transcriptomics to reveal precise functional targets of ILC subsets at different stages of tumor development.

## 7 The relationship between ILCs and the gut microbiota

The gut microbiota refers to the complex community of microorganisms that colonize the intestinal mucosal surface and within the lumen, forming a symbiotic relationship with the host. They are involved in the regulation of the intestinal immune network through interaction with the host via their metabolic products (134). The relationship between ILCs and the gut microbiota centers on their bidirectional regulation: on one hand, microbial metabolites directly influence the differentiation and function of ILCs (135); on the other hand, ILCs shape the composition of the microbiota through the secretion of cytokines. This reciprocal interaction is essential for maintaining intestinal homeostasis (136).

Kairui Mao et al. discovered that gut microbiota-derived SCFAs promote the secretion of IL-22 by ILC3s through a dual mechanism involving the GPR41/HDAC-AhR/HIF1α signaling axis, thereby maintaining intestinal homeostasis (108, 109, 137). The gut microbiota promotes the selective expansion of ILC1s by reducing bile acid levels to inhibit the TET1-DNA hydroxymethylation axis, and maintains the balance of intestinal immunity in adulthood through the regulation of TET1 (138). Segmented filamentous bacteria, upon colonization supported by dietary fiber, activate ILC1s to secrete IFN-γ (139). In the context of *Clostridioides difficile* infection (CDI), antibiotic-induced microbial dysbiosis suppresses ILC2 function and promotes inflammatory skewing toward ILC1s/ILC3s, thereby increasing susceptibility to follow-up infection. Notably, IL-33 therapy can reverse this process and activate ILC2s to secrete the protective factor, AREG (140). The enrichment of *Bacteroides* and *Akkermansia* in the gut microbiota, together with the metabolite 3-hydroxypyridine, synergistically inhibits the proliferation of ILC2s and reduces the secretion of IL-5/IL-13 (141). Gaifem J et al. demonstrated that *Akkermansia muciniphila* and *Parabacteroides distasonis* act synergistically to increase the frequency of ILC3s and enhance the integrity of intestinal epithelial barrier, exerting protective effects against colitis in IBD models (135). In contrast, overactivation of ILC3s by *Escherichia coli* leads to IL-17A pathway-mediated exacerbation of tissue damage (110). Additionally, microbiota-derived tryptophan metabolites regulate the differentiation of ILC3 via the AHR, further influencing their transformation toward a pro-inflammatory phenotype (10, 111).

ILC3s enhance intestinal mucosal barrier function by secreting IL-22, which induces the production of antimicrobial peptides by IECs and promotes tissue repair, thereby directly inhibiting the colonization and invasion of Salmonella (89). Abt MC et al. demonstrated that ILC1s and their secreted pro-inflammatory factors also play a positive role in *Clostridioides difficile*-induced infection (136). Additionally, ILC3s can maintain appropriate gut microbiota composition through ID2-dependent regulation of IL-22, contributing to early resistance against colonization by exogenous pathogens (142).

In summary, the bidirectional regulatory relationship between the gut microbiota and ILCs serves as a critical nexus for maintaining intestinal immune homeostasis and microbial balance (**Figure 5**).

**Figure 5:**
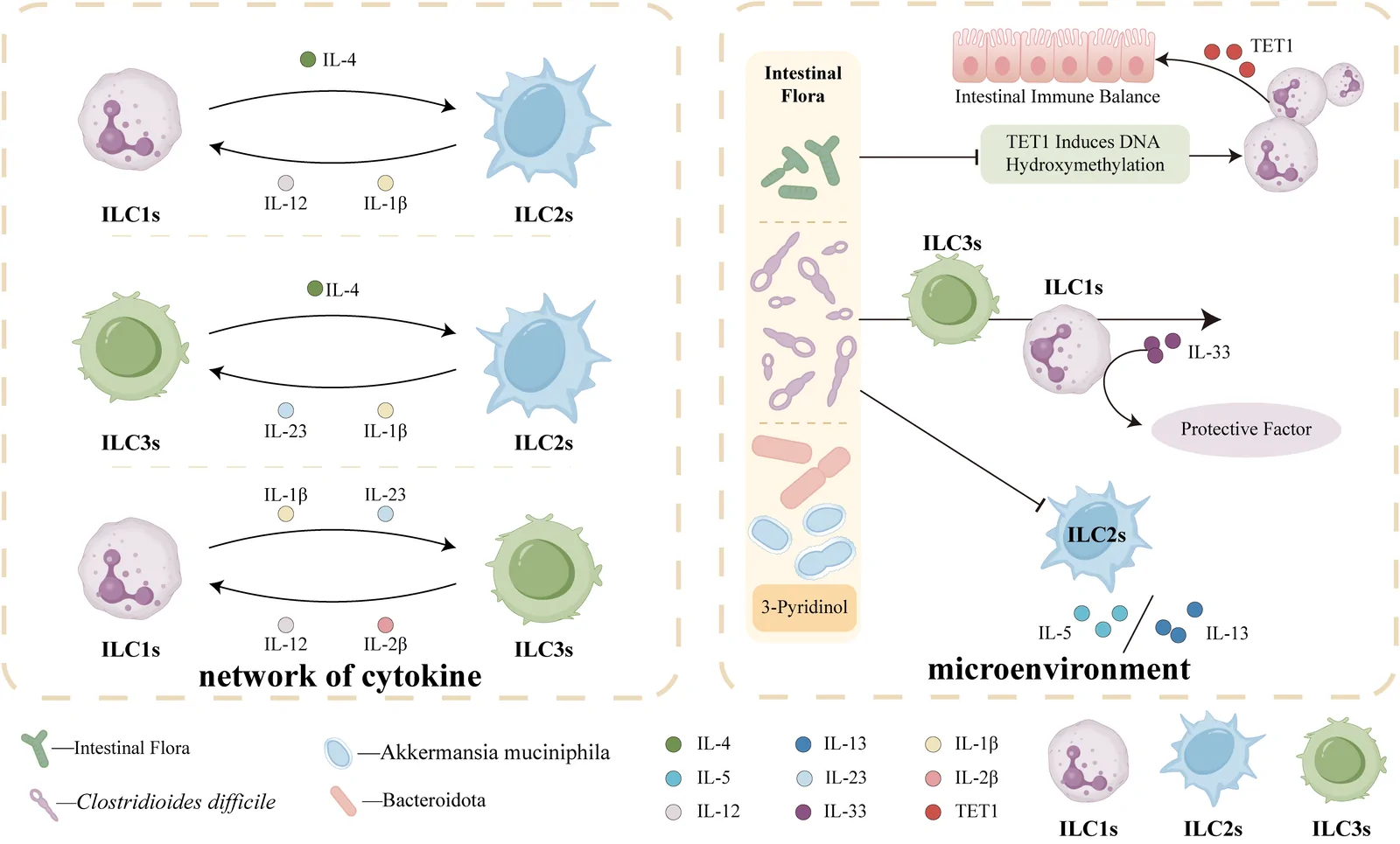
The relationship between ILCs and the gut microbiota.

## 8 Therapeutic potential of targeting ILC subsets and current challenges

ILCs serve as critical regulators of intestinal immune homeostasis, responsible for coordinating tissue repair, microbial defense, and inflammation regulation (143). Evidence indicates that various intestinal diseases, including IBD and CRC, are characterized by abnormal proportions or significant changes in the number of ILCs subsets (100, 126). In patients with Crohn’s disease (CD) and ulcerative colitis (UC), inflamed intestinal mucosa exhibits elevated frequencies of ILC1s and immature NKp44^-^ ILC3s alongside reduced mature NKp44^+^ ILC3s, with peripheral blood demonstrating analogous dysregulation. Biological therapies—including anti-TNF agents, ustekinumab (targeting IL-12/23), and vedolizumab—partially reversed this imbalance through joint reduction of ILC1s (with the notable exception of ustekinumab) and restoration of intestinal NKp44^+^ ILC3s (while anti-TNF concomitantly mobilized circulating NKp44^+^ ILC3s), thereby ameliorating intestinal inflammation, which suggests their mechanistic therapeutic relevance (107) (**Table 2**). These findings underscore the considerable potential of targeting ILC subsets for therapy. Future interventions aimed at ILCs may enable the development of more precise treatments with fewer side effects, offering novel breakthroughs in the management of intestinal diseases. In the future, focusing on the plasticity and tissue specificity of ILCs to develop novel therapies that precisely modulate their functions holds promise for restoring immune homeostasis with greater safety. Nevertheless, challenges remain, including addressing the dual roles of ILCs at different disease stages (balancing their pro-inflammatory and pro-restoring functions) and minimizing the risks of off-target effects in targeted therapies. Overcoming these obstacles will be critical for advancing ILC research from basic science toward clinical translation. However, current research on ILCs still faces several limitations. First, there is significant variability in the reported surface marker profiles used to identify ILC subsets, which has led to contradictory conclusions across studies focusing on the same intestinal disease. Thus, it is essential to integrate current advanced technologies such as single-cell technologies to establish a widely accepted, comprehensive, and accurate lineage map of human and experimental animal intestinal ILCs. Second, most studies on ILCs in the context of intestinal diseases rely heavily on data derived from experimental animals, with a marked lack of clinical evidence, hereby limiting translational potential from basic research to clinical application. Third, currently, most foundational research is largely confined to *in vivo* studies, which may be partly attributed to the low abundance of intestinal ILCs and the technical difficulty in isolating primary cells for *in vitro* experiments. This limitation largely explains why current research on intestinal ILCs has contributed to a predominant focus on phenotypic characterization rather than elucidation of precise regulatory mechanisms. Overcoming this technical barrier is essential to advance related studies in the future.

**Table 2:** The influence of immunotherapy strategies on intestinal mucosa and circulating ILCs.

		Ustekinumab-treated patients	Anti-TNF-treated patients	Vedolizumab-treated patients
ILC1	in the gut mucosa	nd	decrease (106)	decrease (106)
	in circulation	decrease (106)	Decrease (106)/increase (99)	decrease (106)
NKp44+ ILC3	in the gut mucosa	increase (106)	increase (106)	increase (106)
	in circulation	nd	increase (106)	nd
NKp44-ILC3	in the gut mucosa	nd	nd	nd
	in circulation	nd	Increase (106)/decrease (99)	Increase (99, 106)

nd, not determined.

## 9 Discussion

ILCs play a crucial role in the immune system, especially in maintaining immune homeostasis and regulating inflammatory responses. In recent years, an increasing number of studies have revealed the complex roles that ILCs play in intestinal diseases (**Table 3**). ILCs are not only executors of immune responses but also integrators and modulators of information. Within the specific microenvironment of the gut, these cells participate in regulating intestinal immune responses through interactions with the gut microbiota, epithelial cells, and other immune cells. However, in certain gastrointestinal diseases, the activity of specific ILC subsets related to immune overreaction is enhanced, while in other intestinal diseases, their functions may be suppressed. Moreover, the boundary between the beneficial immune functions of ILCs and their involvement in excessive immune responses remains blurred. This complex duality underscores the necessity of being cautious about the divergent findings across studies while interpreting the roles of ILCs in intestinal diseases.

**Table 3:** The types of research references.

	Reference
Studies based on human	(13, 25, 26, 27, 30, 32, 41, 44, 47, 48, 49, 50, 53, 61, 73, 75, 77, 81, 82, 100, 101, 102, 105, 106, 107, 112, 115, 117, 118, 119, 121, 122, 132)
Studies based on murine models	(4, 6, 8, 9, 10, 16, 17, 18, 19, 24, 28, 29, 33, 34, 35, 36, 37, 38, 39, 40, 42, 43, 51, 54, 55, 56, 57, 58, 59, 60, 64, 65, 66, 67, 68, 69, 70, 71, 72, 74, 76, 78, 79, 80, 86, 88, 89, 90, 91, 92, 93, 94, 97, 99, 103, 104, 105, 108, 109, 111, 114, 120, 128, 129, 130, 131, 133, 135, 136, 137, 138, 139, 140, 141, 142)

Controversies in the field have largely focused on phenotypic conversion and functional persistence of ILCs across intestinal disease contexts, yet resolving these disputes requires more stringent criteria for subset identification. Key unresolved issues include the authentic identity of ILC subsets, the evidentiary sufficiency of newly proposed populations such as “ILCregs” as independent lineages, and the standardization of single-cell data annotation.

Conventional ILC1s (cILC1s) and ILC3-derived ILC1-like cells (ex-ILC3s) present substantial phenotypic overlap, as both express T-bet and produce IFN-γ. However, cILC1s typically express CD103, NKG2A, and CD94 while lacking NKp44, CCR6, and CD117, whereas ex-ILC3s in transitional states may display intermediate phenotypes such as CD103^+^CCR6^+^ or CD103^+^CCR6^-^ and retain residual IL-22-producing capacity upon IL-23 stimulation. Discrimination therefore requires integration of developmental origin, epigenetic memory, and surface marker gradients, with lineage tracing serving as the gold standard and epigenetic profiling combined with single-cell RNA velocity analysis as supplementary tools for identifying transitional cells.

The “ILCreg” concept, introduced in 2017, remains contested. Available evidence strongly suggests that IL-10-producing regulatory ILCs in the human intestine represent activated ILC2s or ILC3s that have acquired a reversible regulatory functional state under specific microenvironmental signals such as TGF-β and retinoic acid, rather than an independent developmental lineage. Their lack of stably expressed lineage-determining transcription factors analogous to T-bet, GATA3, or RORγt supports this interpretation. The prevailing consensus thus questions whether a well-defined independent ILCreg lineage exists in the human intestine, and researchers are advised to employ the “ILCreg” designation cautiously. Preferred nomenclature includes “IL-10-producing ILC2/ILC3” or “regulatory ILC2/ILC3 (regILC2/regILC3),” with explicit documentation of induction conditions, potential origins (e.g., ex-ILC2/ex-ILC3), and epigenetic evidence attesting to the plasticity of these functional states.

Standardization of ILC subset identification in single-cell datasets is equally pressing. A recommended workflow proceeds through filtering, stratification, module scoring, manual curation, and cross-dataset integration, establishing a three-tiered marker system with lineage-determining transcription factors (TBX21, GATA3, RORC) as the core layer, functional effector molecules (IFNG, IL5, IL22) as the validation layer, and surface receptors (CD103, PTGDR2, NKp44) as the refinement layer, while accounting for species-specific differences such as the absence of EOMES in human helper-like ILC1s. Practical challenges abound: lineage-determining factors frequently exhibit continuous and co-expression patterns that complicate threshold setting; effector molecule expression is highly stimulus-dependent; lineage tracing, the gold standard, is not feasible in human studies, leaving indirect inference methods with inherent limitations; algorithm selection for cross-dataset integration (Harmony, Seurat CCA) affects sensitivity; and conventional scRNA-seq lacks spatial resolution despite the critical importance of microenvironmental context for ILC function. Automated preliminary screening via SingleR or AddModuleScore should be followed by manual verification of cluster-specific marker co-expression through violin plots and dot plots, with batch correction during cross-dataset integration to accurately capture plasticity trajectories such as ILC3-to-ILC1 conversion.

Discrepancies in published findings extend beyond technical limitations in subset identification and non-standardized annotation to reflect deeper biological realities: the poorly characterized intermediate transitional states inherent to ILC plasticity, and the substantial heterogeneity of the intestinal microenvironment across anatomical regions, disease stages, inflammatory states, and individuals, together with the temporal dynamics of ILC responses. These factors collectively produce divergent functional states of nominally identical subsets across studies, generating apparent contradictions. Advances in single-cell sequencing, spatial multi-omics (spatial transcriptomics, multiplex immunofluorescence), and high-dimensional flow cytometry/mass cytometry now offer means to resolve these dynamics with finer granularity, potentially revealing the network mechanisms through which ILCs operate in intestinal diseases and identifying novel therapeutic targets. Priority directions for future research include precise mapping of ILC subset dynamics and plasticity pathways across diseases; elucidation of signaling pathways governing acquisition or loss of regulatory states; characterization of ILC interactions with epithelial, stromal, neuronal, and microbial compartments; and development of strategies targeting pathogenic ILC states or enhancing regulatory ones. Notably, advances in single-cell sequencing and multi-omics technologies have opened new avenues for in-depth investigation into the dynamic changes of ILCs and their roles within the gastrointestinal microenvironment. These approaches hold great promise for elucidating the complex networks involving ILCs in intestinal diseases and may facilitate the identification of novel therapeutic targets for the prevention and treatment of gastrointestinal disorders. In conclusion, the role of ILCs in intestinal diseases represents a complex and challenging area of research. A deeper understanding of the phenotypic plasticity and functional reprogramming of ILCs not only enhances our knowledge of the regulatory mechanisms of the immune system but also offers novel therapeutic insights for relevant clinical intervention.

In summary, the role of ILCs in intestinal diseases constitutes a complex and challenging area of investigation. A deeper understanding of ILC phenotypic plasticity, functional reprogramming, and their dependence on microenvironmental context and temporal dynamics will not only advance our knowledge of immune regulatory mechanisms but also provide more translationally promising strategies for precision interventions based on immune cell state modulation.
